# Confounder-Invariant Representation Learning (CIRL) for Robust Olfaction with Scarce Aroma Sensor Data: Mitigating Humidity Effects in Breath Analysis

**DOI:** 10.3390/s25226839

**Published:** 2025-11-08

**Authors:** Md Hafizur Rahman, Jayden K. Hooper, Alaa Wardeh, Ashok Prabhu Masilamani, Hélène Yockell-Lelièvre, Jayan Ozhi Kandathil, Mojtaba Khomami Abadi

**Affiliations:** Noze, 4920 Pl. Olivia, Montreal, QC H4R 2Z8, Canada

**Keywords:** aroma sensors, aroma data, confounder invariant learning, representation learning, scarce data, relative humidity, deep learning, autoencoders, generalizability

## Abstract

Confounding factors in olfactory aroma data, such as high humidity levels, substantially affect sensor outputs, masking subtle volatile organic compound (VOC) patterns and hindering generalizable machine learning models. Traditional representation learning methods often require large datasets to mitigate confounder-induced variance, a resource unavailable in specialized sensor applications with limited data. This study presents Confounder-Invariant Representation Learning (CIRL), a method designed to mitigate confounding influences in data-scarce settings by leveraging explicit confounder information, such as relative humidity. CIRL enhances learned representations by reducing confounder effects, improving data purity and model robustness. Applied to three breath aroma datasets—acetone, ketosis, and peppermint-oil breath, all affected by high humidity—CIRL was integrated with standard autoencoder models. Evaluated within the same framework, CIRL improved generalization performance by 10–15% in classification accuracy across all three datasets. These results demonstrate CIRL’s potential to advance reliable artificial olfaction for applications like breath-based diagnostics in challenging real-world conditions.

## 1. Introduction

Digital olfaction systems, or electronic noses (e-noses), have emerged as a promising technology for non-invasive diagnostics by mimicking the human sense of smell to detect patterns of volatile organic compounds (VOCs) [1]. E-noses are based on an array of partially selective sensors with cross-reactivity and use pattern-recognition methods to interpret complex scent mixtures. Several e-nose architectures have been developed, differentiated primarily by their transduction mechanisms. The main types currently used include chemiresistive [2], piezoelectric [3], optical [4], electrochemical [5], bioelectronic [6] or hybrid systems. Chemiresistive e-noses are the most widely employed due to their simplicity, scalability, and compatibility with portable designs. These systems rely on materials whose electrical resistance changes upon VOC adsorption, such as conductive polymers [7], polymer–carbon black composites [8,9], metal-oxide semiconductors [10], or carbon nanostructures like CNTs [11] and graphene [12].

E-nose technology presents the potential to analyze exhaled breath for early detection of diseases such as cancer [13,14] and metabolic disorders [15,16], offering a rapid, cost-effective, and patient-friendly alternative to invasive methods. These systems operate by capturing a characteristic “scentprint” from a sample’s VOC profile that can reflect underlying physiology. However, clinical translation has been limited by the poor reliability of sensor data in real-world conditions. Chemical sensor arrays are highly susceptible to confounding factors such as humidity, temperature gradients, ambient air dilution, and inter-individual variability which distort the VOC scentprint and can degrade classification accuracy. Among these, humidity is dominant in breath analysis because exhaled samples are naturally near saturation (≈95–99% RH at 37 °C). Water molecules compete for adsorption sites, shift surface charge, and alter the dielectric environment of polymer and metal-oxide films, leading to baseline drift, hysteresis, and non-linear responses [17]. The combination of high humidity and chemically complex breath matrices makes it difficult to separate disease-specific VOC signals from background physiological noise.

Addressing these issues requires a multipronged strategy that prioritizes sensor-level robustness and measurement discipline: (i) sensing materials with intrinsic hydrophobicity or selective permeability; (ii) coatings and architectures that mitigate water uptake; (iii) controlled sampling with preconditioning (dew-point control and humidity-filtering materials); (iv) on-board environmental metrology (dedicated humidity and temperature channels) with baseline management; and (v) data-driven compensation suited for clinical workflows. Historically, humidity compensation has progressed from purely hardware solutions (preconditioning chambers, flow/temperature control) to hybrid sensor-algorithmic approaches that combine reference channels, signal normalization, and modest regression models for temperature–humidity correction [17,18,19,20,21,22,23,24,25]. For example, rapid detection systems using baseline manipulation or orthogonal signal decomposition have been proposed to mitigate humidity drift in MOS arrays, improving VOC selectivity in breath samples [18,23]. Algorithmic interference suppression, such as temperature-humidity compensation via regression models, has suppressed environmental noise in e-noses for aroma quality assessment, but these often require large calibration datasets unavailable in scarce breath e-nose dataset scenarios [19,24]. In breath analysis, studies on exhaled VOCs for disease detection (e.g., lung cancer) highlight humidity’s role in masking biomarkers, with compensation via moisture filters or signal normalization achieving partial success but failing under variable humidity levels [20,25]. These methods can substantially reduce environmental variance, but performance can still degrade under rapidly changing humidity, across devices, or when calibration data are scarce.

Transitioning to machine learning, representation learning methods have been applied to e-nose data to learn robust features invariant to confounders, bridging sensor materials and data processing [26]. Domain adversarial networks, inspired by domain adaptation, train models to minimize confounder influence by aligning distributions across humidity domains, as seen in breath VOC classification where adversarial losses suppress humidity-induced variance [27,28]. Variational autoencoders (VAEs) enforce probabilistic disentanglement of latent factors, enabling separation of VOC signals from environmental noise in sensor time-series data [29,30]. However, these methods often assume large datasets for variance capture, limiting applicability to scarce breath aroma data where overfitting exacerbates confounder entanglement [31,32].

Disentangled representation learning has emerged as a promising extension for explicit confounder isolation in sensor applications, though challenges persist in non-linear, materials-specific interactions [26,33]. Hamaguchi et al. [34] proposed a similarity loss-based framework for disentangling factors in image data, but its reliance on pairwise constraints fails to enforce semantic separation in sensor signals with humidity confounders, as critiqued by Locatello et al. [32] for lacking inductive biases in non-linear spaces. Sanchez et al. [35] introduced mutual information maximization for paired data, yet without adversarial mechanisms, it struggles with entangled confounders like humidity that non-linearly interact with VOC responses [36,37]. Denton and Birodkar [38] developed DRNET for video disentanglement using adversarial training to separate content from pose, but its focus on temporal factors overlooks materials-related confounders such as sensor drift in e-noses [39]. Wu et al. [40] used orthogonality constraints in Vector-Decomposed Disentanglement (VDD) to split domain-invariant and domain-specific representations, effective for domain shifts but insufficient for humidity’s subtle, non-linear effects on sensor materials without adversarial debiasing [41]. Cheng et al. [42] proposed Disentangled Feature Representation (DFR) for decoupling class-specific features from variations, showing promise in few-shot tasks but assuming separable confounders, which fails in breath data where humidity tightly entangles with VOCs [27].

Recent surveys underscore these limitations in olfaction contexts, noting the need for confounder-robust methods in scarce data regimes [26,43]. For breath analysis, disentangled adversarial autoencoders have been explored for subject-invariant features in physiological signals, but few target measurable confounders like humidity in aroma sensors [44,45]. Invariant Risk Minimization (IRM) and Domain Separation Networks focus on domain shifts, while Variational Fair Autoencoders address fairness, yet none fully disentangle supervised confounders in materials-constrained e-nose data [28,31].

To address this critical gap, we propose Confounder-Invariant Representation Learning (CIRL), a disentangled autoencoder framework. CIRL is designed to adversarially separate task-relevant VOC features from humidity-related confounders, learning a purified, humidity-invariant representation of the aroma data. CIRL integrates adversarial disentanglement with explicit humidity prediction, purifying VOC latents for robust classification in scarce breath datasets, advancing beyond these approaches by leveraging sensor-specific confounder information [46]. In this paper, the approach was validated on three challenging real-world datasets involving acetone quantification and breath analysis. This demonstrates that CIRL not only successfully isolates humidity information but also significantly improves classification accuracy and robustness, particularly in the data-scarce and high-humidity conditions characteristic of clinical breath analysis. This work represents a significant step toward developing reliable and deployable digital olfaction systems for medical diagnostics.

## 2. Materials and Methods

### 2.1. E-Nose Devices

#### 2.1.1. Chemiresistive Sensing Array Chip

**Figure 1:**
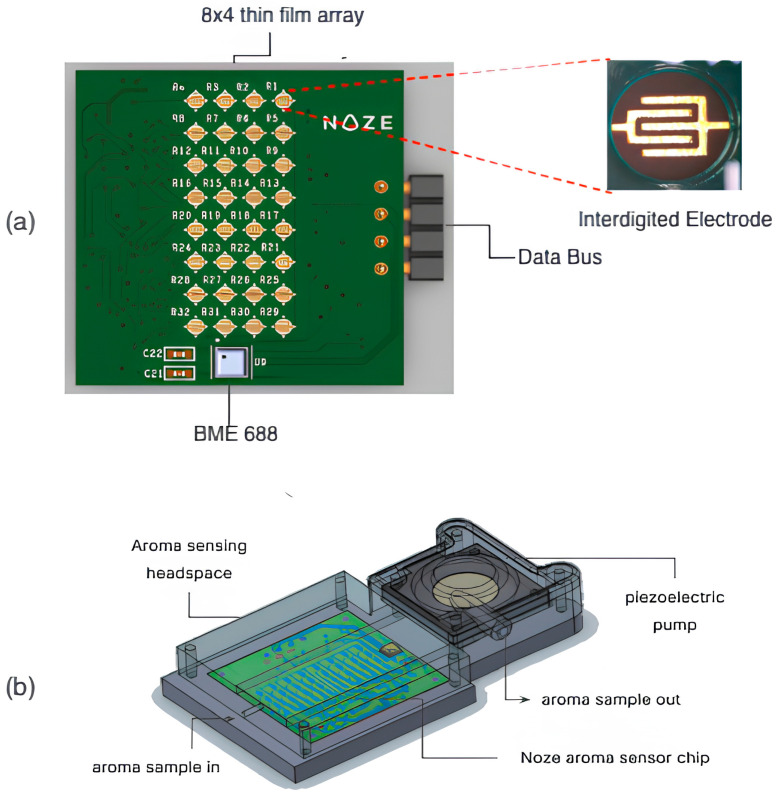
(**a**) The aroma sensor chip used for the method validation experiments, featuring an array of 32 chemiresistive sensing thin films. (**b**) The headspace setup holding the aroma sensor chip, with the piezoelectric pump ensuring control of incoming airflow.

The e-nose devices used in this experiment utilizes the Noze aroma sensor chip (Figure 1a), featuring an array of 32 distinct chemiresistive sensing elements. The substrate for the aroma sensor is fabricated using Electroless Nickel Immersion Gold (ENIG)-plated gold interdigitated electrodes (IDEs) patterned on copper traces over a 1 mm Rogers substrate. Each IDE finger has a width of 100 µm with an inter-electrode gap of 100 µm, and the electrode height is ~12 µm. The chemiresistive sensor thin films are based on proprietary polymer–carbon black (CB) nanocomposites [47], approximately 1 µm thick. The polymer selection follows the functional diversity typically adopted in e-nose designs, covering a wide range of chemical functionalities to generate distinct and complementary sorption patterns, as explained in detail elsewhere [48,49]. A readout circuit measures resistance changes at a sampling rate of 1 Hz, generating a 32-dimensional time-series that serves as the aroma’s unique “scentprint”. To monitor environmental conditions, an off-the-shelf BME688 sensor was integrated to provide real-time temperature and humidity levels in the aroma sample. The aroma sensor chip is enclosed in a specially designed headspace which is filled through a pump for active aroma sampling (Figure 1b).

#### 2.1.2. Vial-Based Aroma Sampler (Noze Inc., Montreal, QC, Canada) Setup

**Figure 2:**
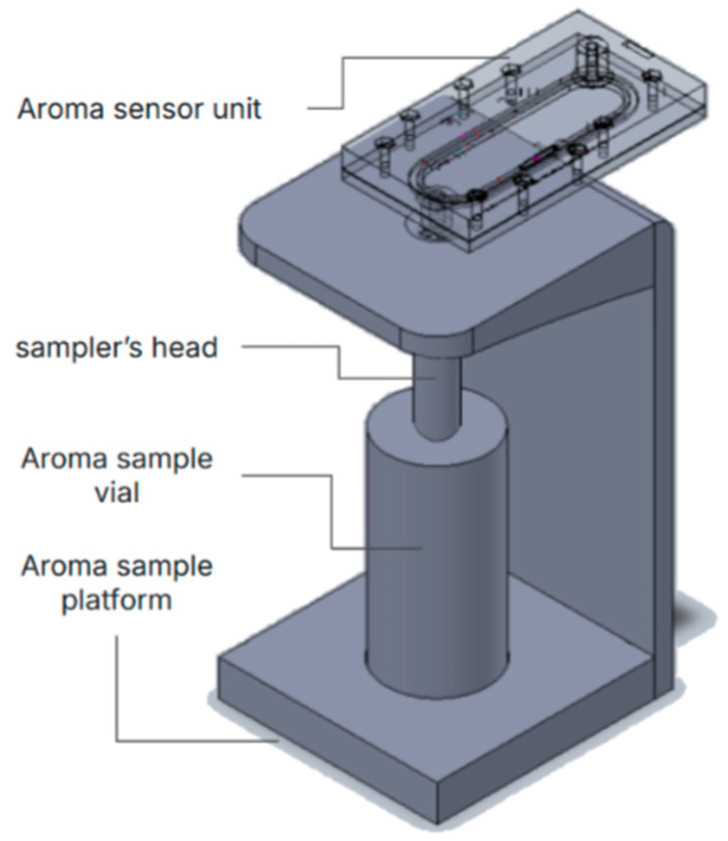
The sampling setup used for the vial-based acetone headspace experiment.

In the first set of experiments, a prototype was designed (Figure 2) in order to expose the e-nose to the headspace of a 20 mL glass vial filled with 10 mL of DI water spiked with small volumes of acetone. The vial headspace is connected to the e-nose headspace via a 5 mm PTFE tube. The pump is operated continuously at a flow rate of 10 mL/min during all the phases of the experiments, while the tube is manually switched between the ambient air and the vial headspace.

The standardized, 3-phase aroma digitization protocol consists of (i) ambient sampling phase: 30 s of sampling air from the ambient as a reference, then (ii) aroma sampling phase: 30 s of sampling the vial’s headspace, followed by (iii) sensor recovery phase: 50 s of sampling from the ambient air again. During the sensor recovery phase, the tube is switched to ambient air in order to clean the sensors by flushing away VOCs from the chip surface; vial pressure changes are negligible due to low flow and large headspace, preventing sample distortion.

#### 2.1.3. Breathalyzer Device Setup

**Figure 3:**
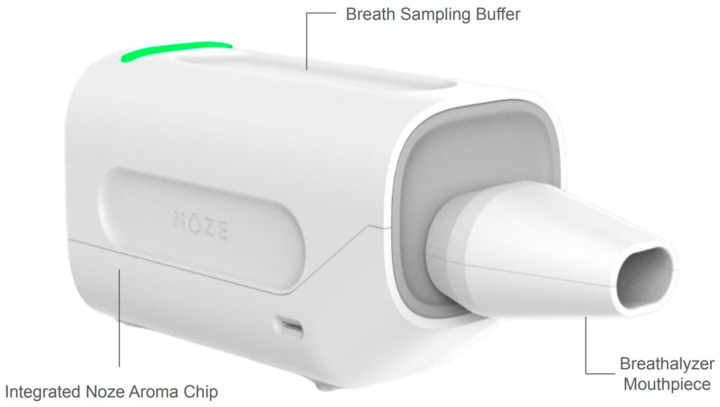
The DiagNoze breathalyzer setup used for the experiments conducted on human breath.

For the two sets of experiments involving breath sampling, samples were collected from participants using the DiagNoze Breathalyzer (Manufactured by Noze Inc., Montreal, QC, Canada). This setup (Figure 3) integrates the digital nose unit in a breath sampling module to detect VOCs in exhaled breath. Participants exhale into a detachable, single-patient-use mouthpiece fitted with a microbial filter, humidity filter, and backflow prevention valve for safety. The breath sampling module has a capnography valve which allows only the alveolar portion, enriched with metabolic VOCs, into an internal buffer chamber which is then directed to the digital nose unit for digitization into an aroma scentprint. The pump operates similarly for controlled flow during sampling phases. The same sampling protocol listed in Section 2.3 was used for all three sets of experiments, in order to ensure consistency.

### 2.2. Description of the Experiments

The CIRL humidity-leveraging approach was experimentally validated in three sets of experiments, using a chemiresistive sensor array deployed in two different setups (Table 1).

#### 2.2.1. Acetone Headspace

In the first set of experiments, the e-nose was exposed to the headspace of a vial of DI water containing varying, precisely quantified amounts of acetone. Six different acetone solutions in 10 mL of water in 20 mL glass lab vials were tested: 0 µL (pure water), 5 µL, 10 µL, 20 µL, 50 µL, and 100 µL, measured using a microsyringe (Hamilton TLC 25 µL).

#### 2.2.2. Ketogenic Breath

In the second set of experiments, the breath of a volunteer (male, age 37) was sampled as they went through four ketogenic diet cycles. Each of the four cycles included two weeks of being on the ketosis diet with a maximum carbohydrates daily intake of 20 g total, and one week of normal carbohydrates-rich diet. A ketosis monitoring breathalyzer (Biosense, Irvine, CA, USA) was used to measure the ketosis state of the volunteer.

#### 2.2.3. Peppermint Breath

The third set of experiments was conducted on volunteers (*n* = 19; 14 male, 5 female; age 24–45) and is based on a benchmarking protocol for breath analysis developed by Henderson et al. [50,51] Breath samples from participants were recorded before and after they ingested a capsule of essential oil of peppermint (Nature’s Way Pepogest).

### 2.3. Confounder-Invariant Representation Learning (CIRL) Method

The CIRL, a deep learning framework designed to explicitly disentangle task-relevant VOC features from confounder-related signal variations (Appendix A), was developed to address the challenge of humidity confounding in breath VOC analysis by e-noses.

#### 2.3.1. Conceptual Framework

The core idea behind CIRL is to learn a latent representation of the sensor data that is split into two distinct parts: one that contains only the information needed to perform the classification task (the VOC fingerprint) and another that captures the information related to the confounder (humidity) (Appendix A.1). To achieve this, the model is trained in an adversarial manner, akin to a two-player game. The main model (the encoder) tries to create a “purified” VOC representation that is completely free of any humidity information. Simultaneously, a second part of the model (the confounder predictor) acts as an adversary, trying its best to predict the humidity level from this “purified” representation. By training the encoder to fool the adversary, it learns to systematically strip out humidity-related features, resulting in a latent space that is invariant to the confounder.

#### 2.3.2. Model Architecture

**Figure 4:**
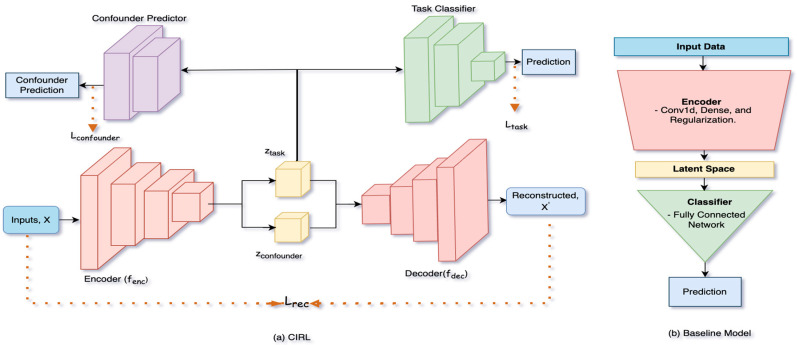
(**a**) Overview of the proposed disentangled autoencoder (CIRL) architecture. The input data $\left(X\right)$ is passed through the encoder $f_{enc}$, which generates two disentangled latent representations: $z_{task}$ and $z_{confounder}$. These representations are used by the decoder $f_{dec}$ for reconstruction, on the other hand $z_{\mathrm{task}}$ used by the task classifier and confounder predictor for their respective outputs. The overall training is governed by the loss functions $L_{rec}$, $L_{\mathrm{task}}$ and $L_{\mathrm{confounder}}$, combined into $L_{\mathrm{total}}$. (**b**) The architecture of the baseline model. The baseline model was designed to isolate the effect of CIRL’s architecture. It uses an encoder and task classifier identical to CIRL’s but lacks the decoder, the split latent space, and the adversarial confounder predictor, mapping all features to a single latent space. This ensures a fair comparison where performance gains can be directly attributed to the proposed disentanglement mechanism.

The CIRL framework is implemented as a disentangled autoencoder with three key components (Figure 4):

1. Encoder ($f_{enc}$): A series of temporal convolutional layers that maps the input sensor data X into two separate latent spaces: a task-relevant space $z_{task}$ and a confounder space $z_{confounder}$.
2. Decoder ($f_{dec}$): A series of transposed convolutional layers that reconstructs the original input signal from both latent spaces, forcing the model to learn a complete representation.
3. Classifier and Confounder Predictor: The task classifier $\left(c\right)$ uses only the purified $z_{task}$ to predict the task label. The confounder predictor $\left(h\right)$ attempts to predict the humidity signal from $z_{task}$.

### 2.4. Training and Optimization

The model is trained by optimizing a composite loss function that balances three objectives (Appendix A.3):

- Reconstruction Loss ( $L_{rec}$): Ensures the decoded signal accurately reconstructs the original input.
- Task Loss ($L_{task}$): Ensures the task-relevant latent space $z_{task}$ is predictive of the target label.
- Confounder Loss ($L_{confounder}$): Used adversarially. While the confounder predictor minimizes this loss to find humidity information, the encoder is trained to maximize it, forcing the encoder to make $z_{task}$ invariant to humidity.

The total loss is formulated as: $L_{total}=\lambda_{rec}L_{rec}+\lambda_{task}L_{task}-\lambda_{confounder}L_{confounder}$

The hyperparameters $\lambda_{rec}$, $\lambda_{task}$, and $\lambda_{confounder}$ govern the balance between reconstruction fidelity, task performance, and confounder invariance in the CIRL framework:

- The parameter $\lambda_{rec}$ emphasizes reconstruction fidelity, where a higher weight (e.g., 1.0–2.0) ensures accurate reconstruction of sensor signals. However overemphasis risks retaining humidity information in $z_{task}$, reducing humidity-invariance representation.
- The parameter $\lambda_{task}$ controls the importance of learning task-relevant attributes, and hence underweighting it can lead to poor task performance.
- The parameter $\lambda_{confounder}$ encourages learning humidity-invariant attributes alongside retaining task-relevant information; however, setting it to an excessive weight (e.g., >0.5) may disrupt task-relevant attribute encoding.

**The training and optimization pseudocode as follows (Algorithm 1):**
**Algorithm 1.** The training and optimization pseudocode**Input:** Data X, confounders C, labels y, initial $\lambda_{rec}$, $\lambda_{task}$, and $\lambda_{confounder}$ Initialize $f_{enc}$, $f_{dec}$, h and c with random weights Initialize an optimization method with a suitable learning rate for $f_{enc}$, $f_{dec}$ and c Initialize a different optimization method with an appropriate learning rate for h
**For each epoch:** $\left(z_{task},\;z_{confounder}\right)$ ← $f_{enc}\left(X\right)$ $\hat{X}$ ← $f_{dec}\left(z_{task},z_{confounder}\right)$ $\hat{C}$ ← $h\left(z_{task}\right)$, $\hat{y}$ ← $c\left(z_{task}\right)$ Compute $L_{rec}$ as a chosen distance metric between *X* and $\hat{X}$ Compute $L_{\mathrm{task}}$ as a selected error measure between *y* and $\hat{y}$ Compute $L_{\mathrm{confounder}}$ as a chosen error measure between *C* and $\hat{C}$

$L_{\mathrm{total}}$ ← $\lambda_{rec}L_{rec}+\lambda_{task}L_{task}-\lambda_{confounder}L_{confounder}$ Update
$f_{\mathrm{enc}}$,
$f_{\mathrm{dec}}$, *c* to minimize $L_{\mathrm{total}}$ with their optimization method Update
*h* to maximize $L_{\mathrm{confounder}}$ with its optimization method and gradient reversal
Optionally adjust $\lambda_{i}$ using $\lambda_{i}\left(t\right)=\frac{\left\|\nabla L_{i}\right\|}{\left\|\nabla L_{total}\right\|}$ **End For**


### 2.5. Data Preprocessing

Before model training, all sensor signals underwent a standardized preprocessing pipeline:

1. Ambient Normalization: Each sensor’s response was normalized using the formula: $x\prime_{i}=\left(x_{i}/median\left(x_{baseline}\right)\right)-1$. This normalization strategy preserves the relative magnitude of sensor responses while compensating for inter-sensor variability and baseline drift.
2. Temporal Sequence Truncation: Input sequences are truncated at the recovery phase terminus plus 60 s, capturing the complete VOC desorption dynamics while eliminating uninformative tail regions. This fixed-window approach ensures consistent temporal context across samples, encompassing baseline (30 s), exposure (30 s), and recovery phases (50 s + 60 s buffer), totaling approximately 170 timesteps at 1 Hz sampling.
3. Zero-Padding: To maintain uniform tensor dimensions required for batch processing in convolutional architectures, sequences shorter than the maximum length are right-padded with zeros post-recovery phase. This post-sequence padding strategy preserves temporal causality and prevents the introduction of artificial signal artifacts during convolution operations, as the padded regions are effectively masked by the learned kernels’ receptive fields.

To address the class imbalance in the ketosis dataset, we used class weighting during training, which adjusts the loss function to give more emphasis to the minority (high-ketone) class.

### 2.6. Experimental Setup and Evaluation

To validate CIRL, we compared its performance against a baseline model. The baseline model consisted of an identical encoder architecture and task classifier but used a single, unified latent space and lacked the decoder and adversarial components (Figure 4b). This ensures that any performance gains are attributable to the disentanglement mechanism.

All experiments were conducted using a 5-fold stratified cross-validation protocol (60% train, 20% validation, 20% test). Hyperparameters for both models (Table 2) were optimized using Bayesian optimization [52] over 50 trials, with the validation macro F1-score as the objective metric.

Models were implemented in TensorFlow 2.18 and trained on NVIDIA A100 GPUs (NVIDIA, Santa Clara, CA, USA). Detailed model architectures are provided in Table 3.

Performance was evaluated using F1-score, precision, recall, and Area Under the Curve (AUC). To directly quantify disentanglement, we also measured the MSE of humidity signal reconstruction from the latent spaces.

## 3. Results

### 3.1. Training Dynamics and Model Convergence

**Figure 5:**
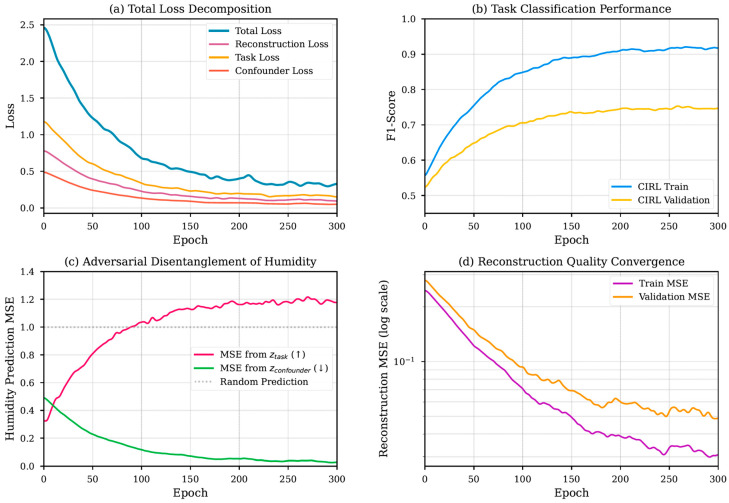
Training dynamics for CIRL on acetone dataset: (**a**) Total loss decomposition showing balanced optimization of reconstruction, task, and adversarial objectives. (**b**) Task classification F1-score demonstrating CIRL’s superiority over baseline. (**c**) Adversarial humidity disentanglement-increasing MSE from confirms successful invariance learning. (**d**) Reconstruction quality convergence on log scale.

Analysis of the training dynamics provides insight into CIRL’s adversarial learning process. Figure 5 illustrates the evolution of the loss components and performance metrics for the acetone dataset. The total loss decomposition (Figure 5a) shows balanced optimization with all component losses converging smoothly. While the confounder loss term decreases as part of the overall optimization, the adversarial dynamics are better understood through the disentanglement metrics in Figure 5c.

The most compelling evidence of disentanglement is shown in Figure 5c. As training progresses, the ability of the adversary to reconstruct the humidity signal from the task space $z_{task}$ deteriorates significantly (MSE increases). Conversely, its ability to reconstruct humidity from the dedicated confounder space $z_{confounder}$ improves (MSE decreases). This clear divergence confirms that humidity-related information is being systematically purged from the task-relevant features and isolated in the confounder space.

### 3.2. Quantitative Evaluation of Disentanglement

To formally validate the disentanglement, we measured the final humidity signal reconstruction error from each latent space after training (Table 4). The results show a stark contrast: the humidity signal could not be accurately reconstructed from $z_{task}$ (MSE > 0.89), confirming that humidity information has been successfully removed. In contrast, the signal could be reconstructed with very high fidelity from $z_{confounder}$ (MSE < 0.05), proving that this information was isolated.

### 3.3. Classification Performance

With disentanglement validated, we assessed its impact on downstream classification performance. As shown in Table 5 (Acetone) and Table 6 (Breath datasets), CIRL consistently and significantly outperformed the baseline model across all tasks.

On the 6-class acetone classification task, CIRL achieved a macro F1-score of 0.75 ± 0.33, a 16% absolute improvement over the baseline’s 0.59 ± 0.04; this improvement was found to be statistically significant (paired *t*-test across CV folds, *p* < 0.01). The gains were most pronounced for the challenging low-concentration classes, where humidity interference is most likely to obscure the weak acetone signal.

On the breath datasets, the improvements were even more dramatic. For the highly imbalanced ketosis dataset, the performance gain was even more pronounced. CIRL elevated the F1-score for high-ketosis detection from a near-random 0.42 (baseline) to a robust 0.88 (paired *t*-test, *p* < 0.01). Similarly, for the peppermint-oil task, CIRL achieved a stable F1-score of 0.74, whereas the baseline model failed to learn a meaningful pattern for the post-ingestion class (0.38 F1) (paired *t*-test, *p* < 0.01).

These results confirm that the gains from disentanglement are not just large, but statistically robust.

### 3.4. Ablation Study

To isolate the contribution of each component in the CIRL framework, we conducted an ablation study (Table 7). The results show a clear progression: adding the reconstruction objective provides a solid performance boost (+9–16% F1). However, the final and most significant gains (+7–16% additional F1) are delivered by the adversarial disentanglement component. This confirms that adversarial invariance is the key innovation responsible for achieving robustness to confounders.

## 4. Discussion

The challenge of detecting trace volatile organic compounds (VOCs) in exhaled breath is underscored by the high-humidity, low-concentration conditions of our experiments. In the ketogenic diet study, the breath acetone levels can vary from 0.5 to more than 40 ppm [53], but the high breath humidity presents a challenge for precise monitoring. Similarly, in the peppermint breath experiment, the breath samples that are recorded at different periods of time after ingestion illustrate the predictable washout profiles of the different peppermint VOCs [50], but the VOCs trace amounts (in the 10 s of ppb) can easily be dwarfed by the high breath humidity content, around 50,000 ppm. The power of CIRL lies in its explicit, supervised disentanglement mechanism. Rather than implicitly hoping a model learns to ignore confounders, CIRL’s adversarial architecture forces the encoder to generate a task-relevant representation, $z_{task}$, that is probably free of humidity information (Appendix A.2). This is empirically validated by our key finding: the humidity signal could not be accurately reconstructed from $z_{task}$ (MSE > 0.89), while being preserved with high fidelity in the dedicated confounder space, $z_{confounder}$ (MSE < 0.05). This answers why CIRL succeeds where other models falter: by actively purging the dominant, non-linear distortions caused by water vapor, it allows the downstream classifier to focus on the subtle, low-magnitude VOC patterns that are otherwise obscured. The performance gains—especially the elevation of the high-ketosis F1-score from a near-random 0.42 to a robust 0.88—demonstrate this principle in action. The baseline model was overwhelmed by the humidity signal, whereas CIRL successfully isolated the weak biomarker signature.

An important finding from our experiments is CIRL’s robust performance across chemically distinct VOCs. The first two experiments targeted acetone, a small, polar, and hydrophilic molecule that is miscible with water. In contrast, the peppermint-oil experiment targeted menthone and menthol, which are larger, terpene-based compounds that are mostly hydrophobic. The fact that CIRL demonstrated consistent and significant performance gains in both scenarios suggests that its mechanism is largely independent of the target analyte’s specific chemical properties. This implies that CIRL works by learning to isolate the signature of water vapor itself, rather than learning the specific, complex interactions between water and a particular VOC. This makes the framework highly generalizable and robust for analyzing diverse chemical mixtures.

The implications of these findings for future clinical e-nose studies are concrete and significant. By effectively neutralizing the impact of humidity, CIRL can achieve the following:

- Improve Reproducibility and Standardization: It reduces a major source of inter-sample and inter-device variability, a critical barrier that has stalled the clinical adoption of e-nose technology.
- Enhance Feasibility of Point-of-Care Screening: By making the device more robust to the uncontrolled ambient humidity of a clinical setting, it lowers the need for complex and costly environmental controls, making widespread deployment more practical.
- Increase Reliability in Longitudinal Studies: By correcting for short-term humidity-induced drift, CIRL can improve the reliability of monitoring disease progression or treatment response over time, where distinguishing true biological change from instrumental variation is paramount. Furthermore, the CIRL framework is sensor-agnostic. While we used a nanocomposite array, the principle can be applied to any e-nose technology (e.g., MOS, CP, QCM) that is susceptible to humidity that can be measured with a dedicated humidity sensor, and/or other measurable confounding factors.

Despite these strengths, we acknowledge certain limitations that open avenues for future research. The current framework was optimized for a single, measured confounder (humidity). In real-world applications, e-nose data are often affected by multiple confounding variables simultaneously, such as ambient temperature and long-term sensor drift due to material aging. Extending CIRL to handle this is not trivial. A potential architectural adjustment could involve a multi-head confounder predictor, where $z_{task}$ is made invariant to a vector of confounders (*C*_1_, *C*_2_, … *C_n_*) by training parallel adversarial discriminators for each. However, this would introduce practical challenges in balancing the multiple adversarial losses and would require a larger $z_{confounder}$ space to capture the joint variance. Future work should therefore focus on extending the CIRL framework to this multi-confounder scenario, perhaps by adding parallel adversarial predictors for temperature. Furthermore, applying this methodology to longitudinal datasets will be crucial for developing models that are robust not only to environmental interference but also to the inevitable effects of long-term sensor drift, thereby enhancing the stability and reliability of e-nose systems in chronic disease monitoring.

## 5. Conclusions

In this work, we introduced and validated Confounder-Invariant Representation Learning (CIRL), a deep learning framework designed to address the critical challenge of humidity interference in electronic nose systems. We demonstrated that by using an adversarial training mechanism, CIRL successfully learns to disentangle task-relevant VOC features from humidity-induced signal variations. Our experiments, conducted on three distinct datasets including challenging real-world breath samples classification, showed that this purification of the feature space enabled significant and consistent improvements in classification performance, with absolute F1-score gains of up to 16% over a comparable baseline model. The framework proved particularly effective in data-scarce and class-imbalanced scenarios (Appendix A.4). Ultimately, this work establishes a viable software-based path toward developing more reliable and deployable digital olfaction systems, helping to bridge the gap between laboratory potential and real-world clinical application.

## Figures and Tables

**Table 1:** Summary of Acetone Headspace and Breath Analysis Datasets used in the experiments.

Dataset	Total Samples	Classes	Key Challenge	Source Device
Acetone Headspace	385	6 levels (0–100 μL acetone)	humidity confounds acetone signal	vial-based headspace sampler (Manufactured by Noze Inc., Montreal, QC, Canada)
Ketogenic Breath	168	low-ketones (112); high-ketones (56)	humidity confounds acetone signals; imbalance	DiagNoze breathalyzer (Manufactured by Noze Inc., Montreal, QC, Canada)
Peppermint Breath	361	pre-ingestion (191); post-ingestion (170)	trace VOC detection amid high humidity; variability	DiagNoze breathalyzer (Manufactured by Noze Inc., Montreal, QC, Canada)

**Table 2:** Hyperparameter configuration determined through Bayesian optimization.

Parameter	Search Range	Baseline	CIRL
Learning Rate	[1 × 10^−4^, 1 × 10^−2^]	1 × 10^−3^	3 × 10^−4^
Batch Size		32	32
λ*_rec_*	[0.5, 2.0]	–	1.0
λ*_task_*	[0.5, 2.0]	–	1.5
λ*_conf_*	[0.1, 0.5]	–	0.3

**Table 3:** Example architectural specifications of the experiment performed on Acetone Headspace Dataset.

Component	Baseline Model	CIRL Model
Encoder	3 Conv1D layers, Filters: [256, 128, 64] Kernel Size: 3, Stride: 2 BatchNorm + LeakyReLU (0.2)	3 Conv1D layers, Filters: [256, 128, 64] Kernel Size: 3, Stride: 2 BatchNorm + LeakyReLU (0.2)
Latent Space	52-dim (unified)	32-dim $z_{task}$ + 20-dim $z_{confounder}$
Decoder		Mirror of the encoder
Task Classifier	2 FC [256, 128] Dropout: 0.3 Input: Full Latent	2 FC [256, 128] Dropout: 0.3 Input: $z_{\mathrm{task}}$
Humidity Predictor		2 FC [128, 256] + 1D TransposedConv Input: $z_{\mathrm{task}}$ Output: Humidity Signal

**Table 4:** Humidity signal reconstruction MSE from the task-relevant vs. confounder-specific latent spaces.

Dataset	MSE from *z_task_*	MSE from *z_conf_*
Acetone Headspace	0.89 ± 0.12	0.03 ± 0.01
Ketogenic Breath	1.23 ± 0.15	0.05 ± 0.02
Peppermint Breath	1.15 ± 0.18	0.04 ± 0.01

**Table 5:** Acetone concentration classification results (6-class).

Concentration	Baseline			CIRL		
	F1-Score	Precision	Recall	F1-Score	Precision	Recall
C0: 0 μL (water)	0.62 ± 0.04	0.65 ± 0.03	0.60 ± 0.05	0.86 ± 0.02	0.88 ± 0.02	0.84 ± 0.03
C1: 5 μL	0.55 ± 0.05	0.58 ± 0.04	0.52 ± 0.06	0.67 ± 0.03	0.69 ± 0.03	0.65 ± 0.04
C2: 10 μL	0.47 ± 0.06	0.50 ± 0.05	0.45 ± 0.07	0.63 ± 0.03	0.65 ± 0.03	0.61 ± 0.05
C3: 20 μL	0.68 ± 0.03	0.70 ± 0.03	0.66 ± 0.04	0.73 ± 0.02	0.75 ± 0.02	0.71 ± 0.03
C4: 50 μL	0.64 ± 0.04	0.66 ± 0.03	0.62 ± 0.05	0.80 ± 0.02	0.82 ± 0.02	0.78 ± 0.03
C5: 100 μL	0.58 ± 0.05	0.61 ± 0.04	0.55 ± 0.06	0.82 ± 0.03	0.84 ± 0.02	0.80 ± 0.03
Macro Average	0.59 ± 0.04	0.62 ± 0.03	0.57 ± 0.05	0.75 ± 0.03	0.77 ± 0.02	0.73 ± 0.03

**Table 6:** Breath VOC detection performance.

Dataset	Model	F1-Score	Precision	Recall	AUC
Peppermint Pre-ingestion	Baseline	0.51 ± 0.05	0.54 ± 0.04	0.48 ± 0.06	0.52 ± 0.04
	CIRL	0.74 ± 0.03	0.76 ± 0.03	0.72 ± 0.04	0.81 ± 0.02
Peppermint Post-ingestion	Baseline	0.38 ± 0.06	0.42 ± 0.05	0.35 ± 0.07	0.46 ± 0.05
	CIRL	0.74 ± 0.03	0.73 ± 0.03	0.73 ± 0.04	0.82 ± 0.02
High Ketosis	Baseline	0.42 ± 0.07	0.45 ± 0.06	0.39 ± 0.08	0.48 ± 0.06
	CIRL	0.88 ± 0.03	0.89 ± 0.02	0.87 ± 0.03	0.93 ± 0.02

**Table 7:** Ablation study demonstrating incremental benefits of CIRL components.

Configuration	Acetone Headspace F1	Ketogenic Breath F1	Peppermint Breath F1
Baseline (single latent)	0.59 ± 0.04	0.60 ± 0.06	0.45 ± 0.05
+Reconstruction loss	0.68 ± 0.03	0.75 ± 0.04	0.61 ± 0.04
+Adversarial training (full CIRL)	0.75 ± 0.03	0.91 ± 0.02	0.74 ± 0.03

## Data Availability

The Acetone Headspace data are available from the corresponding author upon reasonable request. The breath (Ketogenic and Peppermint) datasets are not publicly available due to privacy restrictions, as they contain personally identifiable information.

## Appendix A. Theoretical Framework for CIRL

### Appendix A.1. Problem Formulation and Information-Theoretic Definitions

Consider a breath aroma dataset where X∈X (where X denotes the input data and X its domain space) represents time-series inputs (e.g., impedance responses from an aroma chip), y∈Y (where y is the task label and Y its label space) denotes task labels (e.g., VOC concentrations), and C∈C (where C represents confounders and C their space) captures confounders (e.g., relative humidity). The joint distribution $D=p\left(X,y,C\right)$ (where D is the data distribution and *p*(·) the probability density) often entangles these elements, obscuring the relevant signal from the VOC mixture. Our goal is to learn a latent representation $z=\left(z_{task},z_{confounder}\right)$ (where z is the combined latent vector, split into task and confounder parts), where $z_{task}\in {\mathbb{R}}^{d_{task}}$ (the real-valued vector space of dimension $d_{task}$, a hyperparameter for task-relevant dimensionality) isolates task-relevant features and $z_{confounder}\in {\mathbb{R}}^{d_{confounder}}$ (similarly, dimension $d_{confounder}$ for confounders) captures confounder effects, enabling robust predictions even with limited data.

To formalize this separation, we first need to define the information-theoretic tools that underpin CIRL. Mutual information quantifies the shared information between variables, while conditional mutual information accounts for dependencies given additional context—both are crucial for disentangling confounders.

### Appendix A.2. Disentanglement, Identifiability, and Theoretical Guarantees

**Definition** **A1** **(Mutual Information).** *The mutual information $I\left(A;B\right)$* *(denoted as $I\left(\cdot ;\cdot \right)$, measuring dependency between variables) between two random variables* A and B *measures the reduction in uncertainty about* A *given knowledge of* B*, defined as:* $I\left(A;B\right)=E_{p(A,B)}[log\;log\;\frac{p(A,B)}{p\left(A\right)p(B)}\;]$ *where E* *denotes the expectation (average) over the joint probability distribution* p(A,B), *and p(·)* *represents the probability density function. or equivalently,* $I\left(A;B\right)=H\left(A\right)-H(A|B)$, *where H(·) **is the entropy (uncertainty measure) and* H(·|·) *the conditional entropy.*

**Definition** **A2** **(Conditional Mutual Information).** *The conditional mutual information $I\left(A;B|C\right)$**(dependency given a third variable) measures the remaining mutual information between* A *and B* *given C*, *defined as: $I\left(A;B|C\right)=E_{p(A,B,C)}[log\;log\;\frac{p(A,B|C)}{p(A|C)p(B|C)}\;]$* *or $I\left(A;B|C\right)=H\left(C\right)-H\left(B,C\right)$*, *reflecting the dependence between A* *and* B *conditioned on C*.

With these tools, we can now define what it means for a representation to be disentangled and identifiable in the context of CIRL:

**Definition** **A3**** (Disentangled Representation).** *A representation $z=\left(z_{task},z_{confounder}\right)$* *is disentangled with respect to the task and confounders if* *(i)* $z_{task}$ *is sufficient for predicting* y*, i.e.,* $I\left(z_{task};y\right)\approx I\left(X,y\right)$*, where* $I\left(\cdot ;\cdot \right)$ *denotes mutual information.* *(ii)* $z_{task}$ *is invariant to* C*, i.e.,* $I\left(z_{task};C\right)\approx 0$. *(iii)* $z_{confounder}$ *captures the information in* C*, i.e.,* $I\left(z_{confounder};C\right)\approx I\left(X;C\right)$.

This ensures VOC features are isolated from humidity in breath data.

**Definition** **A4** **(Identifiable Representation).** *A disentangled representation $z=\left(z_{task},z_{confounder}\right)$* *is identifiable if, given the data distribution D, there exists a unique pair of mappings $f_{enc}:X\to {\mathbb{R}}^{d_{task}}\times {\mathbb{R}}^{d_{confounder}}$* *(encoder function $f_{enc}$**) and $f_{dec}:{\mathbb{R}}^{d_{task}}\times {\mathbb{R}}^{d_{confounder}}\to X\left(decoder\cdot function\cdot f_{dec}\right)$* *(decoder function $f_{dec}$* *) (up to permutation and scaling) that satisfy the disentanglement conditions. In practice, this uniqueness supports reliable VOC detection despite humidity variations.*

To achieve this disentanglement and identifiability, certain conditions must hold. We build on prior [54] and adapt it to aroma data scenarios:

**Assumption** **A1** **(Conditional Independence).** *The task labels y and confounders c are conditionally independent given the input* X*, i.e., $p\left(y,C|X\right)=p(y|X)p(C|X)$*.

**Assumption** **A2** **(Sufficient Encoder).** *The encoder $f_{\mathrm{enc}}$* *is sufficiently expressive to capture the information in* X*, i.e., $I(f_{enc}(X);X)\approx I(X;X)$ [55]*.

With these assumptions, we can prove that CIRL learns an identifiable representation:

**Theorem** **A1** **(Identifiability of CIRL Representations).** *Under the above Assumptions (Conditional Independence and Sufficient Encoder), and assuming the confounder predictor $h:{\mathbb{R}}^{d_{task}}\to C$* *is trained to optimality, the CIRL model learns an identifiable representation $z=(z_{task},z_{confounder})$* *such that:* *(i)* $z_{task}$ *is invariant to c, i.e.,* $I(z_{task};C)=0$*.* *(ii)* $z_{task}$ *is sufficient for y, i.e.,* $I(z_{task};y)=I(X;y)$. *(iii)* $z_{confounder}$ *captures c, i.e.,* $I(z_{confounder};C)=I(X;C)$.

**Proof.** The confounder loss $L_{confounder}=E_{\left(X;C\right)\sim D}[l_{confounder}(C,h(z_{task}))]$ is minimized adversarially by maximizing the error of h. At optimality, h cannot predict C from $z_{task}$, implying $I(z_{task};C)=0$. This follows from the data processing inequality [56], if $h(z_{task})$ contains no information about C, then $z_{task}$ is independent of C. The task loss $L_{task}=E_{\left(X,y\right)\sim D}[l_{task}(y,C(z_{task}))]$ ensures that $z_{task}$ retains information about y. By the Sufficient Encoder assumption, the encoder captures all relevant information in X. Since $L_{task}$ optimizes c to predict y, and y is conditionally independent of C (the Conditional Independence assumption), we have $I(z_{task};y)\geq I(X;y)$. The equality holds when $z_{task}$ is a minimal sufficient statistic for y [54]. The reconstruction loss $L_{rec}=E_{x\sim D}[l_{rec}(X,f_{dec}(z_{task},z_{confounder}))]$ ensures that $z_{task}$ and $z_{confounder}$ jointly encode all information in X. Since $z_{task}$ is invariant to C, the remaining information about C must be encoded in $z_{confounder}$. Thus, $I\left(z_{confounder};C\right)=I(X;C)$ [54]. Identifiability follows from the uniqueness of the decomposition under the conditional independence assumption, as shown in [54]. The encoder and decoder are unique up to permutation and scaling, as the loss functions enforce distinct roles for $z_{task}$ and $z_{confounder}$. □

### Appendix A.3. Optimization as an Information Trade-Off

CIRL achieves this separation through a carefully designed optimization process. The total loss combines multiple objectives: $L_{\mathrm{total}}=\lambda_{\mathrm{rec}}L_{\mathrm{rec}}+\lambda_{\mathrm{task}}L_{\mathrm{task}}-\lambda_{\mathrm{confounder}}L_{\mathrm{confounder}}$ (where $\lambda_{\mathrm{rec}}$, $\lambda_{\mathrm{task}}$, and $\lambda_{\mathrm{confounder}}$ are hyperparameters balancing the losses), where the negative $L_{\mathrm{confounder}}$ enforces adversarial invariance, minimizing $I(z_{\mathrm{task}};C)$. This ensures $z_{\mathrm{task}}$ focuses on task-relevant features, while $z_{\mathrm{confounder}}$ absorbs confounder effects, aligning with the identifiability proof.

This optimization can be understood through an information-theoretic lens, balancing retention and invariance:

**Lemma** **A1** **(Information Trade-Off).**
*The CIRL loss: $L_{total}=\lambda_{rec}L_{rec}+\lambda_{task}L_{task}-\lambda_{confounder}L_{confounder}$* *implicitly optimizes the objectives,*
*(i) Maximizing* $I(z_{task},z_{confounder};X)$ *via* $L_{rec}$. *(ii) Maximizing* $I(z_{task};y)$ *via* $L_{task}$. *(iii) Minimizing* $I(z_{task};C)$ *via* $L_{confounder}$*. This trade-off is key for scarce breath data, where retaining VOC info while discarding humidity improves generalization.*

**Proof.** The reconstruction loss $L_{rec}$ minimizes the divergence between x and $\hat{X}=f_{dec}(z_{task};z_{confounder})$ (where $\hat{X}$ is the reconstructed input). For Gaussian noise models, this corresponds to maximizing $I(z_{task},z_{confounder};X)$ [56,57]. The task loss $L_{task}$ optimizes the classifier c (task predictor $c:{\mathbb{R}}^{d_{task}}\to Y$), which maximizes the mutual information $I(z_{task};y)$ by ensuring $z_{task}$ is predictive of y [58]. The adversarial confounder loss $L_{confounder}$ minimizes $I(z_{task};C)$ by training h (confounder predictor $h:{\mathbb{R}}^{d_{task}}\to C$) to fail at predicting C, as shown in the proof of Theorem A1 [27]. □ This aligns CIRL with the Information Bottleneck principle [58], where $z_{task}$ serves as a compressed, confounder-free representation.

### Appendix A.4. Generalization Bound

Finally, we assess CIRL’s generalization to new aroma data, deriving a bound on task error:

**Theorem** **A2** **(Generalization Bound) Let $H_{task}$****(hypothesis class for the task classifier).** *be the hypothesis class of the task classifier $c:{\mathbb{R}}^{d_{task}}\to Y$**, and let $D_{n}={\left\{(X_{i},y_{i},C_{i})\right\}}_{i=1}^{n}$* *(training set of size n**) be a training set of size* n*. Under the Conditional Independence and Sufficient Encoder assumptions, the expected task error* $E_{\left(X,y\right)\sim D}[l_{task}(y,c(z_{task}))]$ *(where $l_{task}$* *is the task-specific loss) is bounded as:* $E_{l_{task}}\leq +O(\sqrt{\frac{VC\left(H_{task}\right)loglog\;n+loglog\;(1/\delta )\;}{n}})$ *(where O(·)* *denotes big-O notation, bounding the error term asymptotically as* n *grows), with probability at least 1−δ* *(confidence parameter* δ*), where ${\hat{L}}_{task}$* *is the empirical task loss, and $VC(H_{task})$* *(Vapnik–Chervonenkis dimension measuring class complexity) is the VC-dimension of $H_{task}$**. This bound highlights CIRL’s efficiency in small* n *regimes, common in breath sensor data. Proof: Since $z_{task}$* *is invariant to C* *(Theorem A1), the task classifier* c *operates on a confounder-free representation. The expected task error depends only on the complexity of $H_{task}$* *and the sample size n**. Applying standard VC-dimension bounds [59],* *the generalization gap is:* $E\left[l_{task}\right]-\leq O(\sqrt{\frac{VC\left(H_{task}\right)loglog\;n\;+loglog\;(1/\delta )\;}{n}})$ *The conditional independence of y* *and C* *given X* *(Assumption A1) ensures that confounder variations do not inflate the generalization error, completing the proof.*

This bound confirms that CIRL’s invariance to confounders reduces the risk of overfitting to spurious correlations, enhancing robustness across diverse conditions.
